# Supplementary material for: FibreCastML: an open web platform for predicting electrospun nanofibre diameter distributions for biomedical applications
Source: Front Bioeng Biotechnol. 2026 Feb 18;14:1713804. doi: 10.3389/fbioe.2026.1713804 (PMC12957282; doi:10.3389/fbioe.2026.1713804)
Supplement: Supplementary file 1 [file DataSheet1.zip › Supplementary material- FibreCastML/Supplementary_Material.docx]

FibreCastML: An Open Web Platform for Predicting Electrospun Nanofibre Diameter Distributions for Biomedical Applications

Elisa Roldán^1^*, Kirstie Andrews^1^, Stephen M. Richardson^2^, Reyhaneh Fatahian^1^, Glen Cooper^3^, Rasool Erfani^1^, Tasneem Sabir^4^, Neil D. Reeves^5^

^1^ Department of Engineering, Faculty of Science & Engineering, Manchester Metropolitan University, Manchester M1 5GD, UK

^2^ Division of Cell Matrix Biology and Regenerative Medicine, School of Biological Sciences, Faculty of Biology, Medicine and Health, The University of Manchester, Stopford Building, Oxford Rd, Manchester, M13 9PT

^3^ School of Engineering, University of Manchester, Manchester, M13 9PL, UK

^4^ Manchester Fashion Institute, Faculty of Arts & Humanities, Manchester Metropolitan University, Manchester M15 6BG, UK

^5^ Lancaster Medical School, Faculty of Health and Medicine, Lancaster University, Lancaster LA1 4YW, UK

*** Correspondence:**[Elisa.Roldan-Ciudad@mmu.ac.uk](mailto:Elisa.Roldan-Ciudad@mmu.ac.uk)

Keywords: Artificial Intelligence, Machine Learning Models, Electrospinning, Nanofibres, Open Access Web Application, Meta-Analysis.

Supplementary Material

# Results for each ML model for the 16 polymers

Files, within the folders, are all the results obtained with all the ML models for each polymer except for PET, PEEK, PLA and γ-PGA and with the following conditions: concentration of the solution 12%, needle gage 20, rotational speed 2000 rpm, voltage 25 kV, flow rate 1 ml/h and distance between needle and collector 11 cm.

PET, PEEK, PLA and γ-PGA were not included due to file limits for supplementary material submission. Please, contact the correspondence author to request these files.

Excel files include tabs with:

- Summary of the experiments and recommendations of solvents and percentages.
- Out of range electrospinning parameters with user value and maximum and minimum values reported in the database.
- Plot of Observed vs Predicted values.
- Diameter distribution predictions.
- Metrics (R^2^, RMSE and MAE).
- Regression coefficients (if available).
- Variable importance.
- SHAP values.
- Correlation matrix.
